# Extra-Limites

**Published:** 1835-01-01

**Authors:** 


### EXTRA-LIMITES.

THE

### BATHS OF PFEFFERS,

[IN THE COUNTRY OF THE GRISONS.]

By JAMES JOHNSON M.D.

PHYSICIAN EXTRAORDINARY TO THE KING,

&c. &c. &c.

AMONG the strange places into which man has penetrated in search of treasure or health, there is probably not one on this earth, or under it, more wonderful than the BATHS of PFEFFERS, situated in the country of the Grisons, a few miles distant from the Splugen road, as it leads from Wallenstadt to Coire. They are little known to, and still less frequented by the English; for we could not learn that any of our countrymen had visited them during the Summer of 1834.

Having procured five small and steady horses accustomed to the locality, a party of three ladies and two gentlemen* started from the little town of Ragatz on a beautiful morning in August, and commenced a steep and zig-zag ascent up the mountain, through a forest of majestic pines and other trees. In a quarter of an hour, we heard the roar of a torrent, but could see nothing of itself, or even its bed. The path, however, soon approached the verge of a dark and tremendous ravine, the sides of which were

* Mr. and Miss Hayward, Mrs. and Miss Johnson, and myself.

composed of perpendicular rocks several hundred feet high, and at the bottom of which the TAMINA, a rapid mountain torrent, foamed along in its course to the valley of Sargans, there to fall into the upper Rhine. The stream itself, however, was far beyond our view, and was only known by its hollow and distant murmurs. The ascent, for the first three miles, is extremely fatiguing, so that the horses were obliged to take breath every ten minutes. The narrow path, (for it is only a kind of mule-track,) often winded along the very brink of the precipice, on our left, yet the eye could not penetrate to the bottom of the abyss. After more than an hour of toilsome climbing, we emerged from the wood, and found ourselves in one of the most picturesque and romantic spots that can well be imagined. The road now meanders horizontally through a high, but cultivated region, towards the village of Valentz, through fields, gardens, vineyards, and meadows, studded with chaumiers and chalets, perched fantastically on projecting ledges of rock, or sheltered from the winds by tall and verdant pines. The prospect from Valentz, or rather from above the village, is one of the most beautiful and splendid I have any where seen in Switzerland. We are there at a sufficient distance from the horrid ravine, to contemplate it without terror, and listen to the roaring torrent, thundering unseen, along its rugged and precipitous bed. Beyond the ravine we see the monastery and village of Pfeffers, perched on a high and apparently inaccessible promontory, over which rise alpine mountains, their sides covered with woods, their summits with snow, and their gorges glittering with glaciers. But it is towards the East that the prospect is most magnificent and varied. The eye ranges, with equal pleasure and astonishment, over the valley of Sargans, through which rolls the infant Rhine, and beyond which the majestic ranges of the Rhetiken Alps, ten thousand feet high, rise one over the other, till their summits mingle with the clouds. Among these ranges the SCESA-PLANA, the ANGSTENBERG, the FLESCH, (like a gigantic pyramid,) and in the distance the Alps that tower round Feldkirck are the most prominent features. During our journey to the Baths, the morning sun played on the snowy summits of the distant mountains, and marked their forms on the blue expanse behind them, in the most distinct outlines. But, on our return, in the afternoon, when the fleecy clouds had assembled, in fantastic groups, along the lofty barrier, the reflections and refractions of the solar beams threw a splendid crown of glory round the icy heads of the Rhetian Alps—changing that "cold sublimity" with which the morning atmosphere had invested them, into a glow of illumination which no pen or pencil could portray. To enjoy the widest possible range of this matchless prospect, the tourist must climb the peaks that overhang the village, when his eye may wander over the whole of the Grison Alps and valleys, even to the lake of Constance.

From Valentz we turned abruptly down towards the ravine, at the very bottom of which are the BATHS OF PFEFFERS. The descent is by a series of acute and precipitous tourniquets, requiring great caution, as the horses themselves could hardly keep on their legs, even when eased of their riders. At length we found ourselves in the area of a vast edifice, resembling an overgrown factory, with a thousand windows, and six or seven stories high. It is built on a ledge of rock that lies on the left bank of the TAMINA torrent, which chafes along its foundation. The precipice on the opposite side of the Tamina, and distant about fifty paces from the mansion or rather hospital, rises five or six hundred feet, as perpendicular as a wall, keeping the edifice in perpetual shade, except for a few hours in the middle of the day. The left bank of the ravine, on which the hospital stands, is less precipitous, as it admits of a zig-zag path to and from the Baths. The locale, altogether, of such an establishment, at the very bottom of a frightful ravine, and for ever chafed by a roaring torrent, is the most singularly wild and picturesque I had ever beheld; but the wonders of Pfeffers are not yet even glanced at.

From the western extremity of this vast asylum of invalids, a narrow wooden bridge spans the Tamina, and by it we gain footing on a small platform of rock on the opposite side. Here a remarkable phenomenon presents itself. The deep ravine, which had hitherto preserved a width of some 150 feet, contracts, all at once, into a narrow cleft or crevasse, of less than 20 feet, whose marble sides shoot up from the bed of the torrent, to a height of four or five hundred feet, not merely perpendicular, but actually inclining towards each other, so that, at their summits, they almost touch, thus leaving a narrow fissure through which a faint glimmering of light descends, and just serves to render objects visible within this gloomy cavern. Out of this recess the Tamina darts in a sheet of foam, and with a deafening noise reverberated from the rocks within and without the crevasse. On approaching the entrance, the eye penetrates along a majestic vista of marble walls in close approximation, and terminating in obscurity, with a narrow waving line of sky above, and a roaring torrent below! Along the southern wall of this sombre gorge, a fragile scaffold, of only two planks in breadth, is seen to run, suspended—as it were—in air, fifty feet above the torrent, and three or four hundred feet beneath the crevice that admits air and light from Heaven into the profound abyss. This frail and frightful foot-path is continued (will it be believed?) nearly *half a mile* into the marble womb of the mountain! Its construction must have been a work of great difficulty and peril; for its transit cannot be made even by the most curious and adventurous travellers, without fear and trembling, amounting often to a sense of shuddering and horror. Along these two planks we crept or crawled, with faltering steps and palpitating hearts. It has been my fortune to visit most of the wonderful localities of this globe, but an equal to this I never beheld.

"Imagination, (says an intelligent traveller,) the most vivid, could not portray the portals of Tartarus under forms more hideous than those which Nature has displayed in this place. We enter this gorge on a bridge of planks (pont de planches) sustained by wedges driven into the rocks. It takes a quarter of an hour or more to traverse this bridge, and it requires the utmost precaution. It is suspended over the Tamina, which is heard rolling furiously at a great depth beneath. The walls of this cavern, twisted, torn, and split (les parois laterales contournée, fendues, et dechirées) in various ways, rise perpendicular, and even incline towards each other, in the form of a dome; whilst the faint light that enters from the portal at the end, and the crevice above, diminishes as we proceed;—the cold and humidity augmenting the horror produced by the scene. The fragments of rock sometimes overhang this gangway in such a manner, that the passenger cannot walk upright:—At others, the marble wall recedes so much, that he is unable to lean against it for support. The scaffold is narrow, often slippery; and sometimes there is but a single plank, separating us from the black abyss of the Tamina.* He who has cool courage, a steady eye, and a firm step, ought to attempt this formidable excursion (épouvantable excursion) in clear and dry weather, lest he should find the planks wet and slippery. He should start in the middle of the day, with a slow and measured step, and without a stick. The safest plan is to have two guides supporting a pole, on the inside of which the stranger is to walk."

We neglected this precaution, and four out of the five pushed on, even without a guide at all. At forty or fifty paces from the entrance the gloom increases, while the roar of the torrent beneath, reverberated from the sides of the cavern, augments the sense of danger and the horror of the scene. The meridian sun penetrated sufficiently through the narrow line of fissure at the summit of the dome, to throw a variety of lights and of shadows over the vast masses of variegated marble composing the walls of this stupendous cavern, compared with which, those of Salsette, Elephanta, and even Staffa, shrink into insignificance. A wooden pipe, which conveys the hot waters from their source to the baths, runs along in the angle between the scaffold and the rocks, and proves very serviceable, both as a support for one hand while pacing the plank, and as a seat, when the passenger wishes to rest, and contemplate the wonders of the cavern. At about one-third of the distance

* "Le pont est etroit, souvent glissant, et quelquefois on n'est separé que par une seule planche du noir abîme de la TAMINA."

inward, I would advise the tourist to halt, and survey the singular locality in which he is placed. The inequality of breadth in the long chink that divides the dome above, admits the light in very different proportions, and presents objects in a variety of aspects. The first impression which occupies the mind is caused by the cavern itself, with reflection on the portentous convulsion of Nature which split the marble rock in twain, and opened a gigantic aqueduct for the mountain torrent.* After a few minutes' rumination on the action of subterranean fire, our attention is attracted to the slow but powerful operation of water on the solid parietes of this infernal grotto. We plainly perceive that the boisterous torrent has, in the course of time, and especially when swelled by rains, caused wonderful changes both in its bed and its banks. I would direct the attention of the traveller to a remarkable excavation formed by the waters on the opposite side of the chasm, and in a part more sombre than usual, in consequence of a bridge that spans the crevice above, and leads to the Convent of Pfeffers. This natural grotto is hollowed out of the marble rock to the depth of 30 feet, being nearly 40 feet in width, by 26 feet in height. It is difficult not to attribute it to art; and, as the whole cavern constantly reminds us of the Tartarean Regions, this beautifully vaulted grotto seems to be fitted for the throne of Pluto and Proserpine—or, perhaps, for the tribunal of Rhadamanthus and his brothers of the Bench, while passing sentence on the ghosts that glide down this Acheron or Cocytus—for had the TAMINA been known to the ancient poets, it would assuredly have been ranked as one of the rivers of Hell.

One of the most startling phenomena, however, results from a

* It is surprising that the author of the "Voyage Pittoresque en Suisse," and even Dr. Ebell, should have been led into the monstrous error of imagining that the torrent of the Tamina had, in the course of ages, hollowed out of the marble rock this profound bed for itself. We might just as well suppose, that the bed of the Mediterranean had been scooped out by the waters of the Hellespont, in their way from the Black Sea to the Atlantic. The mountain was rent by some convulsion of Nature, and apparently from below upwards, as the breadth, at the bed of the Tamina, is far broader than the external crevice above.

perspective view into the cavern, when about midway, or rather less, from its portal. The rocky vista ends in obscurity; but gleams and columns of light burst down, in many places, from the meridian sun, through this "palpable obscure," so as to produce a wonderful variety of light and shade, as well as of bas-relief, along the fractured walls. While sitting on the rude wooden conduit before alluded to, and meditating on the infernal region upon which I had entered, I was surprised to behold, at a great distance, the figures of human beings, or thin shadows (for I could not tell which), advancing slowly towards me—suspended between Heaven and earth—or, at least, between the vault of the cavern and the torrent of the Tamina, without any apparent pathway to sustain their steps, but seemingly treading in air, like disembodied spirits! While my attention was rivetted on these figures, they suddenly disappeared; and the first impression on my mind was, that they had fallen and perished in the horrible abyss beneath. The painful sensation was soon relieved by the reappearance of the personages in more distinct shapes, and evidently composed of flesh and blood. Again they vanished from my sight; and, to my no small astonishment, I beheld their ghosts or their shadows advancing along the opposite side of the cavern! These, and many other optical illusions, were caused, of course, by the peculiar nature of the locality, and the unequal manner in which the light penetrated from above into this sombre chasm.

Surprise was frequently turned into a sense of danger, when the parties, advancing and retreating, met on this narrow scaffold. The "laws of the road" being different on the Continent from those in Old England, my plan was to screw myself up into the smallest compass, close to the rock, and thus allow passengers to steal by without opposition. We found that comparatively few penetrated to the extremity of the cavern and the source of the Thermæ—the majority being frightened, or finding themselves incapable of bearing the sight of the rapid torrent under their feet, without any solid security against precipitation into the infernal gulf. To the honour of the English ladies, I must say that they explored the source of the waters with the most undaunted courage, and without entertaining a thought of returning from a half-finished tour to the regions below.*

Advancing still farther into the cavern, another phenomenon presented itself, for which we were unable to account at first. Every now and then we observed a gush of vapour or smoke (we could not tell which) issue from the further extremity of the rock on the left, spreading itself over the walls of the cavern, and ascending towards the crevice in the dome. It looked like an explosion of steam; but the roar of the torrent would have prevented us from hearing any noise, if such had occurred. We soon found, however, that it was occasioned by the rush of vapour from the cavern in which the thermal source is situated, every time the door was opened for the ingress or egress of visitors to and from this natural vapour-bath. At such moments the whole scene is so truly Tartarean, that had Virgil and Danté been acquainted with it, they need not have strained their imaginations in portraying the ideal abodes of fallen angels, infernal gods, and departed spirits, but painted a HADES from Nature, with all the advantage of truth and reality in its favour.

Our ingress occupied nearly half an hour, when we found ourselves at the extremity of the parapet, on a jutting ledge of rock, and where the cavern assumed an unusually sombre complexion, in consequence of the cliffs actually uniting, or nearly so, at the summit of the dome. Here, too, the TAMINA struggled, roared, and foamed through the narrow, dark, and rugged gorge with tremendous impetuosity and deafening noise, the sounds being echoed and reverberated a thousand times by the fractured angles and projections of the cavern. We were now at the source of the THERMÆ. Ascending some steps cut out of the rock, we came to a door, which opened, and instantly enveloped us in tepid steam. We entered a grotto in the solid marble, but of what dimensions

* This has not always been the case. The talented authoress of "Reminiscences of the Rhine," &c. appears to have lacked courage for this enterprise, though her beautiful daughters advanced to the further extremity of the gorge.

we could form no estimate, since it was dark as midnight, and full of dense and fervid vapour. We were quickly in an universal perspiration. The guides hurried us forward into another grotto, still deeper in the rock, where the steam was suffocating, and where we exuded at every pore. It was as dark as pitch. An owl would not have been able to see an eagle within a foot of its saucer eyes. We were told to stoop and stretch out our hands. We did so and immersed them in the boiling—or, at least, the gurgling, source of the Pfeffers. We even quaffed at this fountain of Hygeia.

Often had we slept in damp linen, while travelling through Holland, Germany, and Switzerland. We had now, by way of variety, a waking set of teguments saturated with moisture *ab interno,* as well as *ab externo,* to such an extent, that I believe each of us would have weighed at least half a stone more at our exit than on our entrance into this stew-pan of the Grison Alps.

On emerging into the damp, gellid, and gloomy atmosphere of the cavern, every thing appeared of a dazzling brightness after our short immersion in the Cimmerian darkness of the grotto. The transition of temperature was equally as abrupt as that of light. The vicissitude could have been little less than 50 or 60 degrees of Fahrenheit in one instant, with all the disadvantage of dripping garments! It was like shifting the scene, with more than theatrical celerity, from the Black Hole of Calcutta to Fury Beach, or the snows of Nova Zembla. Some of the party, less experienced in the effects of travelling than myself, considered themselves destined to illustrate the well-known allegory of the discontented—and that they would inevitably carry away with them a large cargo of that which thousands come here annually to get rid of—Rheumatism. I confess that I was not without some misgivings myself on this point, seeing that we had neither the means of changing our clothes nor of drying them—except by the heat of our bodies in the mountain breeze. The Goddess of Health, however, who is nearly related to the Genius of Travelling, preserved us from all the bad consequences, thermometrical and hygrometrical, of these abrupt vicissitudes.*

We retrograded along the narrow plank that suspended us over the profound abyss with caution, fear, and astonishment. The TAMINA seemed to roar more loud and savage beneath us, as if incensed at our safe retreat. The sun had passed the meridian, and the gorge had assumed a far more lugubrious aspect than it wore on our entrance. The shivered rocks and splintered pinnacles that rose on each side of the torrent, in gothic arches of altitude sublime, seemed to frown on our retreating footsteps—while the human figures that moved at a distance along the crazy plank, before and behind us, frequently lost their just proportions, and assumed the most grotesque and extraordinary shapes and dimensions, according to the degree of light admitted by the narrow fissure above, and the scarcely discernible aperture at the extremity of this wonderful gorge. The TAMINA, meanwhile, did not fail to play its part in the gorgeous scene—astonishing the eye by the rapidity of its movements, and astounding the ear by the vibrations of its echoes. It seemed to growl more furiously as we receded from the depths of the crevasse.

At length we gained the portal, and, as the sun was still darting his bright rays into the deepest recesses of the ravine, glancing from the marble rocks, and glittering on the boiling torrent, the sudden transition from Cimmerian gloom to dazzling day-light, appeared like enchantment. While crossing the trembling bridge, I looked back on a scene which can never be eradicated from my memory. It is the most singular and impressive I have ever beheld on this globe, and compared with which, the BRUNNENS are "bubbles" indeed !†

* This circumstance illustrates, in a very remarkable manner, the effects of passing from a hot, or vapour-bath, into cold air or water. The immunity is nearly certain. The hotter the medium from which we start into the cold, the less danger there is of suffering any inconvenience. This principle in Hygiene is more understood than practised. It will be adverted to farther on.

† Lest I should be suspected of exaggeration, in this account of the Baths of

While examining the waters, the baths, and the internal economy of the vast VALETUDINARIUM that stands in this savage locality, the bell announced the approach of the second, or superior dinner, which happened that day to be rather later than usual. The SALON, overlooking the torrent of the Tamina, was soon replenished with guests of the better order; the canaille, or swarm of inferior invalids having dined two hours or more previously, in the common SALLE A MANGER. It needed but little professional discrimination to class and specify them. The majority proclaimed the causes of their visits to the Pfeffers. Rheumatism, scrofula, and cutaneous diseases, formed the prominent features in this motley assemblage. Invalids, with chronic complaints, real or imaginary, such as abound at all watering-places, foreign and domestic, were mingled in the group; while a small portion, including our own party, evinced any thing but corporeal ailments—unless a "CANINE APPETITE," at a genuine German *table d'hôte* may be ranked among the evils to which English flesh is heir.—Some monks, from the neighbouring monastery, (to which the Baths belong,) took rank, and indeed precedence, in this small division. The mountain breeze and fervid sun of the Convent of Pfeffers had

---

Pfeffers, I shall here introduce a short extract from "REMINISCENCES OF THE RHINE, &c." by Mrs. Boddington—a work eulogized to the skies in the Edinburgh Review, and its author represented (and, I understand, deservedly) as a lady of very superior talents and of strict veracity. After some slight notice of the Bath-house, Mrs. B. proceeds thus :—

"Behind rolls the stormy Tamina, hemmed in at one side by the dark Bath-house and the impending cliffs, while, on the other, a giant wall of perpendicular rock, starting up daringly, and shutting out the world—almost the light of Heaven—closes up the scene. Our guide proposed that we should visit the mineral springs that boil up from the depth of an awful cavern, several hundred paces from the Bath-house. A bridge, thrown from rock to rock, crosses the flood, and a narrow ledge of planks, fixed, I know not how, against the side of the rock, and suspended over the fierce torrent, leads through a long, dark chasm to the source. I ventured but a little way; for, when I found myself on the terrifying shelf, without the slightest ballustrade, and felt it slippery, from the continual spray, and saw nothing between us and the yawning gulf, to which darkness, thickening at every step, gave increased horror, I made a few rapid reflections on foolhardiness, and retreated."

bronzed them with much of that nut-brown complexion, which travelling exercise in the open air had conferred on their British visiters; while their sleek cheeks, and portly corporations proved, almost to a demonstration, that the holy fathers descended into the profound ravine of the Tamina to give their benediction to the waters, rather than to drink them—and to add a sacred zest to the viands of the REFECTORY, by the alacrity with which they swallowed them. Their appearance illustrated the truth of the adage—"What will not poison will fatten."

Among the "miseries of human life" might be ranked that of dining, or rather starving, at a German TABLE D'HÔTE—and that, too, in the midst of plenty! It is in such a place that the paradox is explained—"inopem me copia fecit." Sir F. Head has remarked that "the dish that is not acid is sure to be oily." If this were all, we should have small reason to complain. The misfortune is, that not only oils and acids are liberally distributed among his messes, by that infernal agent, the MAITRE DE CUISINE, but every loathsome ingredient that the three kingdoms of Nature can furnish, is crammed into every pot and saucepan in his subterranean dominion. Some philosophers have endeavoured to distinguish man from other animals, and elevate him on the scale of created beings, on account of his *cooking* propensities. I think they entitle him to an additional seven years in PURGATORY, if there be such a place, as our Catholic brethren affirm there is! One thing is clear, however,—that he is punished here below for the crimes which he commits against Nature, by "torturing dishes from their native taste," and mingling all unutterable things in that box of Pandora—his accursed culinary cauldron!

The succession is not less abhorrent to the English palate than the composition of continental dishes. It is generally believed that animal and vegetable food is designed to be eaten together; otherwise Nature would have furnished one side of the mouth with incisors and the other with grinders. In the "continental system" they take a very different view of things. When the vegetables (rather less than half-boiled, and swimming in oil) are on the table, there is no animal food—none, at least, that has not undergone more transubstantiations than Vishnou, and more metamorphoses than are recorded by Ovid. When meat smokes on the board, the vegetables have disappeared! The animal that was browsing or bleating on the mountains, the preceding day, and slaughtered in the night, is burnt to a cinder, or boiled till little more than bones and sinews are left:—In either case, it is some degrees harder and tougher than well tanned sole-leather. As for poor chanticleer, his ablation from the roost—decapitation in the court-yard—auto da fé in the kitchen—dissection in the salle à manger—and sepulture in some dark recess of a German stomach, occupy about three quarters of an hour—the five acts of the tragedy being often enacted *after* the soup has gone its round of the table d'hôte! If the uninitiated Briton sometimes screws his courage up to make an attack on one of those petty fortresses of filth, called "MADE DISHES"—or if he endeavours to stifle the cravings of Nature on sour bread, sour krout, or sour wine, he stands a fair chance to be visited with colic, if not cholera, before the day is over. Placed thus between Scylla and Charybdis—between the tortures of hunger and the terrors of poison, an OASIS in the desert does sometimes greet his eye—a good substantial dish of capon, veal, or mutton. By an instinctive impulse, he brandishes his couteau, or solicits to be *helped* by a brother guest. But the fate of Tantalus is his doom. Just as the prize appears to be within his grasp, it vanishes, with as much celerity as the dishes of poor Sancho did by the conjuror's wand, in the Island of Barataria! The malicious waiter, aware of John Bull's propensities, never takes his eye from the savoury viand till he snatches it off the table, for dissection at the side-board! It is two to one that John Bull never tastes the desired fare. It is handed round to every one, before the *disjecta membra* reach him—if they ever do reach him, which is very problematical! Many a time have I seized the dish at the same moment with the waiter, and captured the prize by an unequivocal threat to chop off two or three of his fingers with my knife, if he persisted in his unhallowed "ABDUCTION,"

Long experience has taught me, that the best plan for an Englishman, whose stomach does not measure three feet in circumference, and who does not possess some secret antidote against all kinds of poisons, is to secure his place at the TABLE D'HÔTE, and, when the soup comes in, to take a walk of full an hour round the town, and then come back to his place—when he may probably find a dish of some kind of animal food, biped or quadruped, with sour bread, on which he may dine. The "vin ordinaire" is, of course, ordinary destruction to all stomachs which have not capacity for a pint of oil to qualify a quart of acid.

The foregoing sketch is not drawn from the ordinary of the Pfeffers—where, indeed, we had better fare than in many places of higher pretensions—but will apply very generally to the Continent. I am well aware that great numbers of my countrymen have become *acclimaté* (if I may use the expression) to foreign cookery—or, more properly speaking, *denaturalized*, as to every thing which they put into their stomachs. By such folks I have been often asked—"how is it that the people of the Continent live and thrive on the provender which you condemn?" My answer has been very short—and I have never received a satisfactory rejoinder. They do *not* live and thrive on the cookery which they use. On the contrary, they wither and die on it. The bills of mortality, in the most favoured parts of the Continent, as compared with the same gloomy registers in England, prove, beyond contradiction, the shorter range of existence enjoyed by the inhabitants of the former, notwithstanding their advantages in respect of climate:—while the unhealthy aspects, the stunted growth, and the large proportion of deformities, that meet the eye and attract the notice of English travellers in every part of Europe, attest the deleterious agency of some general cause on the human frame. As that agency can hardly be sought either wholly, or even principally, in the climate, the soil, the air, or the water, (excepting, of course, certain malarious and goitrous localities in Italy and the Alps,) we have fair reason to attribute much of the curtailment of life and deterioration of health to the denaturalization of their food by complicated cookery—to their inordinate addiction to tobacco—to malpropre habits—and to the quality of their drink. If oily, acid, or rancid dishes, elaborated "de omnibus rebus et *quibusdem aliis*,"—half-boiled vegetables—meat just killed and then cinderized—with sour wine, be wholesome and nutritious, then the people of the Continent ought to live to the age of the Antediluvians.

Another fallacious argument has been adduced in favour of continental cookery and continental habits: namely, that the English enjoy good health while travelling, or even sojourning there. This may be true to the full extent, without invalidating the arguments adduced above. The English owe this improvement of health to climate, to change of air and scene, to the exercise of travelling, to earlier hours than they kept at home—and perhaps, in some degree, to the excitement resulting from novelty, and intercourse with strangers. I maintain that their health is neither improved nor sustained by the adoption of continental habits in eating, drinking, smoking, and some others which I shall not describe.

### THE

### WATERS OF PFEFFERS.

The Waters of Pfeffers have neither taste, smell, nor colour. They will keep for ten years, without depositing a sediment, or losing their transparency. But we are not to infer that they are destitute of medicinal powers, because they possess no sensible properties. In their chemical composition, they have hitherto shewn but few ingredients; and those of the simpler saline substances, common to most mineral springs.* It does not follow, however, that they contain no active materials because chemistry is not able to detect them. Powerful agents may be diffused in waters, and which are incapable of analysis, or destructible by the process employed for that purpose. The only sure test is experience of their effects on the human body. It is not probable that the Baths of Pfeffers would have attracted such multitudes of invalids, annually, from Switzerland, Germany, and Italy; and that for six centuries, if their remedial agency had been null or imaginary.† Their visiters are not of that fashionable class, who run to watering-places for pleasure rather than for health—or, to dispel the vapours of the town by the pure air of the coast or the country. Yet, as human nature is essentially the same in all ranks of society, I have no doubt that much of the fame acquired by the Baths of Pfeffers, has been owing to the auxiliary influence of air, locality, change of scene, moral impressions, and the peculiar mode of using the waters. Their temperature—100° of Fahren.—certain physical phenomena which they evince, and the nature of the diseases

* In an old account of the baths we find the following passage :—"The water of these baths is extremely clear, without taste or smell. It bears with it the most subtle spirits of sulphur, nitre, vitriol, and divers metals—amongst others, GOLD."

† In many people they produce slight vertigo—in more, they act freely on the bowels. They were discovered in the 12th century, by two chasseurs from the neighbouring monastery, who were seeking birds' nests in the ravine of the which they are reported to cure, leave little doubt in my mind that their merits, though overrated, like those of all other mineral springs, are very considerable.

The disorders for which they are most celebrated, are rheumatic and neuralgic pains, glandular swellings, and cutaneous eruptions. But they are also resorted to by a host of invalids afflicted with those anomalous and chronic affections, to which nosology has assigned no name, and for which the Pharmacopœia affords very few remedies. As the Baths belong to the neighbouring Convent of Pfeffers, and, as the holy fathers afford not only spiritual consolation to the patients, but medical assistance in directing the means of cure, there is every reason to believe, or, at least, to hope, that the moral, or rather divine influence of Religion co-operates with mere physical agency, in removing disease and restoring health.

The Waters of Pfeffers are led from their sombre source in the cavern, along the narrow scaffold before described, into a series of Baths scooped out of the rocky foundation of this vast hospital, each bath capable of accommodating a considerable number of people at the same time. The thermal waters are constantly running into and out of the baths—or rather through them, so that the temperature is preserved uniform, and the waters themselves in a state of comparative purity, notwithstanding the numbers immersed in them. The Baths are arched with stone—the window to each is small, admitting little light, and less air;—and, as the doors are kept shut, except when the bathers are entering or retiring, the whole space not occupied by water, is full of a dense vapour, as hot as the Thermæ themselves. The very walls of the baths are warm, and always dripping with moisture. Such are the SUDATORIA in which the German, Swiss, and Italian invalids indulge more luxuriously than ever did the Romans in the Baths of Caracalla. In these they lie daily, from two, to six, eight, ten—and sometimes sixteen hours!* The whole exterior of the body is thus soaked, sof-

---

Tamina. For a long time they could only descend to these baths by means of ropes; but at length human ingenuity formed zig-zags along the rocks. As if every thing relating to these waters should partake of the wonderful, it may be mentioned that they begin to flow in May, when the Summer is approaching—are at their acmé when the skies are fervid and the land parched with thirst, yielding 1500 pints of water every minute—and cease entirely in September, when the rains begin to fall, and the mountain streams to pour freely along every declivity!

* A German writer informs us that the country people stay in these Baths tened—parboiled; while the interior is drenched by large quantities swallowed by the mouth—the patient, all this while, breathing the dense vapour that hovers over the baths. The Waters of Pfeffers, therefore, inhaled and imbibed, exhaled and absorbed, for so many hours daily, must permeate every vessel, penetrate every gland, and percolate through every pore of the body. So singular a process of human maceration in one of Nature's caldrons, conducted with German patience and German enthusiasm, must, I think, relax many a rigid muscle—unbend many a contracted joint—soothe many an aching nerve—clear many an unsightly surface—resolve many an indurated gland—open many an obstructed passage—and restore many a suspended function. The fervid and detergent streams of the Pfeffers, in fact, are actually turned, daily and hourly, through the Augean stable of the human constitution, and made to rout out a host of maladies indomitable by the prescriptions of the most sage physicians. The fable of Medea's revival of youthful vigour in wasted limbs is very nearly realized in the mountains of the Grisons, and in the savage ravine of the Tamina. Lepers are here purified—the lame commit their crutches to the flames—the tumid throat and scrofulous neck are reduced to symmetrical dimensions—and sleep revisits the victim of rheumatic pains and neuralgic tortures.

That many circumstances, connected with the singular locality of the Pfeffers, conduce to their medicinal reputation, there can be little doubt. The Baths themselves, though at the bottom of a ravine, nearly a thousand feet deep, are yet at a considerable height above the neighbouring valley, and very far above the level of the ocean. The air feels peculiarly light and pure, even in the depth of the gorge; while the surrounding precipices and lofty mountains must preserve a remarkable equilibrium of temperature. The sun can penetrate the profundity of the ravine only during a few hours in the middle of the day; and, the sojourners can easily defend themselves from his rays within the walls of this vast sudatorium—or in the cool and gloomy cavern itself. The tempest may roll, the thunders may roar, and the lightnings may play round the lofty alpine peaks; but the profound depth of the ravine maintains its sombre serenity of atmosphere unchanged, and the whole locality looks like a little colony that had sunk from the surface of the

---

from Saturday night till Monday morning. "Tous les Samedis on voit accourir à Pfeffers une multitude de gens des campagne voisines, et ils restent dans le bains jusqu'au Lundi matin pour provoquer la sueur."

upper world, and was only reminded of its existence by the distant war of the elements.

When rains descend into the ravine, the valetudinarians have ample space for exercise under the arcades of the building, or in its spacious "*salles a manger.*" When the weather is fine, there is a terrace in the open air, cut out of the rock close to the Baths, for such as are incapable of much exertion. To those, however, who are able to scale the neighbouring heights, is opened a fund of pleasure and health, such as no place that I have visited on the face of this globe, can present. On the right bank of the Tamina, a staircase is hewn out of the solid marble, by which you ascend to a beautiful little plateau, on which is built the convent, as well as the village of Pfeffers. This table-land is in the form of a triangle, two sides of which are almost perpendicular precipices of nearly a thousand feet—one overhanging the Tamina—the other overlooking the valley of Sargans, through which meanders the upper Rhine. The third side connects this elevated plain with one of the most celebrated Alps of the Grisons—the GALANDA. The monks, in all ages, have evinced their taste in the selection of healthy as well as beautiful sites for their monasteries and convents. The plateau of Pfeffers is most delightfully situated, under the shelter of the GALANDA and other mountains in its rear, and with the romantic valley of Sargans beneath it in front. The ascent to the summit, or *Belvidere of the Galanda,* on this side of the ravine, is a work of labour; but the lover of magnificent scenery would be repaid by one of the most splendid prospects in the world, while the hypochondriacal invalid would, most assuredly, throw off his load of "blue devils," and imaginary ills, before he got half way to the apex of this gigantic pyramid. The chain of the Rhetian Alps rises like a wall before him—the lake of Wallenstadt, with its stupendous and impending scenery, is under his feet—the lake of Constance is in the distance—and a sea of Alps encompasses him on every side.

Invalids of weaker powers, and less *ambitious views,* may mount, almost entirely on mules or small horses, from the western bank of the Tamina—namely, from the Baths, by the romantic village of Valenz, to the mountains that tower over the hamlet, where they will enjoy a prospect little inferior to that which is seen from the GALANDA, and where the sublime and beautiful are scattered in the most bountiful profusion.*

---

* It is equally curious and interesting to observe the series of gradations and changes that present themselves to the eye of the spectator, while standing on

If we consider attentively the remarkable process of bathing, already described—the equable temperature maintained in the ravine—the moral impressions made on the mind of the stranger by the stupendous and romantic scenes around him—the opportunities, and even the inducements, for every species of exercise, from the slow saunter on the level terrace, to the laborious ascent of the cloud-capt Alp—and, lastly, the invigorating influence of the mountain breeze, after protracted immersion in hot water, and long inhalation of tepid vapour—we can scarcely doubt that all these moral and physical agencies combined, must produce very remarkable effects on the human constitution—and those of a very beneficial kind, particularly in certain maladies.

---

an eminence of five or six thousand feet, and in the vicinity of the high Alps. First, the cap of dazzling and unsullied snow, crowning each mountain-top 'in frigid majesty:'—then the naked and primœval granite, starting out through the thinner coats of snow—a little lower down we see specks of scanty vegetation, preserving a miserable and precarious existence amidst storms and avalanches—then the stunted pine, extracting nutriment from the crevices of the rocks—next in succession, we see small pieces of pasturage, maintaining the goat, with its outlaw, the chamois, and presenting the first and worst of human habitations—the Chalet. Descending still lower, the dark "piney forest" contrasts deeply with the masses of "unfathom'd snows" that hang over it, and seems to stand as the barrier between the region of desolation and that of fertility. Now, the Chaumieres or Swiss cottages supersede the chalets, or goatherds' huts, perched on ledges of rocks, and surrounded by meadows, corn-fields, gardens, and even vineyards;—with cattle grazing, shepherds tending their flocks, and peasants labouring in every kind of rural avocation. From the region of eternal snow, down to the sunny vales of the Alps, we see the glittering glaciers wedged in the deep ravines, and slowly descending in rivers of solid ice, each disgorging from its dark recesses a rapid and roaring torrent, the noisy herald of the Alps, announcing their contributions to the mighty ocean. Lastly, the eye rests on the tranquil and glassy lake, the mirror of the mountains, reflecting from its polished surface the hoary peak and frowning cliff—the verdant field and gloomy forest—the solitary hut and smiling cottage—the foaming cataract and fearful precipice—all the materials and features, in short, of the magnificent amphitheatre. The contemplative spectator beholds, with equal delight and astonishment, another Heaven and another Earth depicted ten thousand feet beneath him, illustrating, and infinitely surpassing, the beautiful description of the poet—Prior:—

"As when some smooth expanse receives, impressed,
Calm Nature's image on its wat'ry breast;—
Down bend the banks, the trees depending grow,
And skies beneath, with answering colours glow."

It is clear, however, that there are many complaints to which the Baths of PFEFFERS might prove injurious. In pulmonary affections of all kinds, warm baths are more than doubtful—they are generally prejudicial. The afflux of blood to the surface, while in the bath, must be followed by more or less of efflux from the periphery to the centre of the body—and then the weak organ will experience more injury than benefit from the operation. Besides this, there is a certain degree of re-action that follows all baths, both hot and cold—and this re-action or excitement almost always aggravates the symptoms of chronic inflammation, or organic disease of internal structures. Chronic hepatitis may form an exception sometimes. The excitement of the *tepid* bath on the skin generally increases the secretion of bile, and in that way relieves a congested liver. But, even here, the bath should never be more than tepid.

The same observations apply to all organic affections of the heart. The tide of the circulation, in such cases, should never be accelerated by either warm or cold bathing—or by the exercise of climbing heights, in such localities as the PFEFFERS. I have seen, in my wanderings on the Continent, many invalids incautiously sent to drink and bathe in various medicinal waters, and where injury would almost inevitably be the result.

In determinations (as they are called) to the head—in chronic affections of the membranes, of the vessels, or of the substance of the brain, hot or cold baths are decidedly contra-indicated, and for the reasons already adduced.

As people with acute diseases are never sent to such places as these, it may seem unnecessary to allude to them here; but I cannot help taking this opportunity of cautioning against a practice by no means uncommon in this country—namely, the employment of warm, and even hot, baths in acute rheumatism. I can safely declare that I never yet saw any good effects from such procedure ;—but, on the contrary, that I have very generally observed an augmentation of the fever—or, what is worse, an increased tendency to translation—not merely from joint to joint, but from the surface to some internal organ, especially the heart. I have been long in the habit, while investigating hypertrophy of the heart, succeeding acute rheumatism, to inquire respecting the treatment of the original disease; and I have found that, in more than three-fourths of these cases, the hot bath had been employed to relieve the pains of the limbs. Acute rheumatism is a specific, and not a common inflammation. It is not to be cured by general and local bleeding, like other topical phlegmasiæ. The blood, indeed, will be found highly inflamed; but that does not authorize venesection in this particular case, no more than the same phenomenon would in pregnancy. Acute rheumatism is a very manageable disease, if baths and blood-letting are left alone, in general, and calomel and opium given, with colchicum and saline aperients. Warm evaporating lotions to the parts inflamed are infinitely better than leechings and baths.

I doubt the utility of warm baths in acute inflammation of internal structures generally—and in many of them, where they are sometimes employed, I am confident they are detrimental. It is by no means uncommon to place a patient, labouring under acute hepatitis, in a warm bath after bleeding. It is hazardous to employ this measure *before* the inflammation is checked, and it is unnecessary *afterwards*. The same practice is often pursued, and always with risk, in pneumonia and carditis. Nothing would induce me to order the warm-bath in either of these complaints. Inflammations of the peritoneum and of the urinary organs, including, of course, the kidneys, are those in which I have observed most benefit, and least danger, from the warm-bath. But even in these, very copious bleeding should precede it—the bowels being well cleared—and the secretions rendered as healthy as possible. There are very few other internal inflammations, where I would venture on the warm-bath.

But there is a long catalogue of chronic disorders, to which THERMAL MEDICINAL WATERS, both internally and externally applied, prove extremely useful—especially when aided by the moral and physical circumstances adverted to in this paper, and which exist, in greater or less abundance, at most of the watering-places, in England and on the Continent. Thermal waters act in three principal ways on the human machine:—1st, through the medium of *sensation*, on the nervous system—2nd, through the agency of *temperature*, on the vascular system—and 3rd, by means of their chemical contents, on the secretory and excretory organs. In most chronic complaints, and especially in rheumatism, gout, cutaneous defædations, neuralgia, dyspepsia, glandular swellings, and visceral obstructions, there is pain, uneasiness, or discomfort of some kind, which, indeed, constitutes the chief grievance of the individual. It is no unimportant matter to soothe these sufferings, during the process employed for their cure. The warm bath effects this purpose in an eminent degree, through its agency on the sentient extremities of the nerves distributed over the surface of the body. There is an extensive chain of sympathies established between the skin and the internal viscera ; and, through the medium of this channel, agreeable sensations excited on the exterior, are very often communicated to the central organs and structures themselves. Even in this way, torpid secretions are frequently roused into activity and improved in quality, while the secretory apparatus itself is relieved from a host of painful feelings.

The agency of thermal waters on the vascular system is of the utmost importance. Although the temperature of the blood is 98° of Fahrenheit, the surface of the body, when not fevered, is very many degrees below that point. The warm bath, therefore, when about blood-heat, attracts a strong tide of circulation to the surface, and thus liberates internal organs, for a time, from a congestive state of their vessels. This determination to the surface augments the cutaneous exhalation, and, by a well-known reflex sympathy, increases the secretion of the great glandular viscera of the interior—more especially the liver. Even the gentle and alternate flux and reflux of the circulation, from the interior to the exterior, and vice versâ, produce very beneficial effects, in constitutions where the balance of the circulation is broken in a variety of ways, and where several secretions and excretions are vitiated, by stagnation in some cases, and by inordinate action in others.

The chemical agency of mineral waters is not to be overlooked. They contain, in all probability, many ingredients which we cannot detect—and many known agents, which we cannot imitate by artificial combinations. This is proved by every day's observation. Thus, the saline aperient materials, in mineral waters, will produce ten times more effect than the identical materials, artificially dissolved and commixed. The same is true with respect to the chalybeate springs. A grain of iron in them is more tonic than 20 grains, exhibited according to the Pharmacopœia. It is on these accounts that a course of the saline aperient waters, followed by the light chalybeates, as at Ems and other places, combined with the various moral and physical auxiliaries which I have described, may and do work wonders in many chronic maladies.

It is, however, in that extensive class of human afflictions termed nervous, dyspeptic, and hypochondriacal, that a journey to the Baths of Pfeffers offers strong temptations, and very considerable hopes of amendment. To hypochondriacs especially I would recommend this tour. Let them get sea-sick in the Batavier, mud-sick in the Maaes, and dyke-sick in Holland :—let them then ascend the Rhine, amid all the bustle of steamers and hotels—and wind through the romantic scenery of that noble river. They may visit the Brunnens of Nassau—the shopocracy of Frankfort—the clean, dull towns of Darmstadt and Carlshrue—the old red Castle of Heidelberg—the fairy land of Baden Baden—the prosperous town of Offenburg—the Black Forest—the Falls of the Rhine—the Lake of Wallenstadt, presenting the most splendid lake scenery in Switzerland—and, lastly, the BATHS OF PFEFFERS. Let them be enjoined by their physician to penetrate the gorge of the Tamina, and drink and perspire at the source of the waters in the rock, as the *sine qua non* of cure—let them be conjured to mount the GALANDA, where there is a specific AIR for the removal of low spirits—and then, if their "BLUE DEVILS" are not drowned in the Pfeffers, or blown away on the Alps—they had better jump into the *Tamina*—for their case is hopeless!

But if they experience, as I think they will, the most beneficial consequences of the discipline I have recommended, then I would advise them to prosecute their tour of health still farther. They are now in the vicinity of one of the most magnificent of the Alpine passes—the SPLUGEN. In their way thither, they thread the mazes of the VIA MALA, one of the wonders of the world—where they view, with terror, the infant Rhine struggling through gorges little inferior to that of the Tamina—and over which they pass three times, with the river rolling and roaring 300 feet beneath them.* Descending from the sublime and dreary heights of the Splugen, they behold, with delight and wonder, the road winding down to fair Italy, like a serpent coiled along the rugged steeps of the mountain. Traversing the lake of Como in the steamer, they may wander round the romantic shores of Lugano—embark on the Lago Maggiore, and land on the Boromeo Isles—return by the Simplon, St. Bernard, or Cenis, and penetrate through the centre of Switzerland, back to the Rhine—or across through dull France, to their native shore—ALL IN TWO MONTHS.

---

* See Dr. Beattie's inimitable delineation of the VIA MALA, in "*Switzerland illustrated*"—a work unequalled for the eloquence of the text, the beauty of the plates, and the fidelity of the descriptions.

